# Definitions of unfavorable surgical outcomes and their risk factors based on disability score after spine surgery for lumbar spinal stenosis

**DOI:** 10.1186/s12891-020-03323-0

**Published:** 2020-05-08

**Authors:** Gang-Un Kim, Jiwon Park, Ho-Joong Kim, Feng Shen, Jaewoo Cho, Bong-Soon Chang, Choon-Ki Lee, Heoung-Jae Chun, Jin S. Yeom

**Affiliations:** 1 Spine Center and Department of Orthopaedic Surgery, Seoul National University College of Medicine and Seoul National University Bundang Hospital, 166 Gumiro, Bundang-gu, Sungnam 463-707 Republic of Korea; 2 Department of Orthopaedic Surgery, Hanil General Hospital, 308 Uicheon-ro, Dobong-gu, Seoul, 01450 Republic of Korea; 3 Department of Orthopaedic Surgery, Seoul National University College of Medicine and Seoul National University Hospital, 28 Yeonkeon-dong, Chongro-gu, Seoul, 110-744 Republic of Korea; 4 Department of Mechanical Engineering, Yonsei University, Seodaemun-gu, Seoul, 03722 Republic of Korea

**Keywords:** Lumbar spinal stenosis, Patient-reported outcomes, Minimal clinically important difference, Spine surgery

## Abstract

**Background:**

Risk factors for unfavorable surgical outcomes are dependent on the definitions of the unfavorable surgical outcomes. The aims of this study were to compare risk factors for each unfavorable surgical outcome according to two different definitions of “unfavorable” surgical outcomes after surgery for lumbar spinal stenosis (LSS) as well as compare the clinical course from the preoperative period to 3 years postoperatively between cases with favorable and unfavorable outcomes according to the two different definitions.

**Methods:**

Overall, 295 patients who underwent spine surgery for LSS and a follow-up evaluation at 3 years postoperatively were enrolled and divided into favorable and unfavorable groups, based on two different definitions for unfavorable surgical outcomes, as evaluated at 12 months postoperatively: the patient-reported outcome (PRO) and minimal clinically important difference (MCID) methods. In the PRO method, patients with a postoperative Oswestry Disability Index (ODI) score > 22 were considered as having an “unfavorable” outcome, whereas in the MCID method, those with a postoperative ODI score that changed < 12.8 points from the preoperative value were classified as having an “unfavorable” outcome. As a primary outcome, risk factors for unfavorable surgical outcomes according to each definition were investigated at 12 months postoperatively.

**Results:**

In the PRO method, female sex (*P* = 0.011; odds ratio (OR): 2.340), elementary school attainment (vs. university attainment; *P* = 0.035; OR: 2.875), and higher preoperative ODI score (*P* = 0.028; OR: 2.340) were associated with higher odds for an unfavorable surgical outcome. In the MCID method, a higher preoperative ODI score was associated with higher odds (*P* <  0.001; OR: 0.920) of a favorable surgical outcome. In the PRO method, the favorable outcome group demonstrated significantly lower visual analog scale for back and leg pain and lower ODI scores than the unfavorable outcome group at 3 years postoperatively, whereas in the MCID method, clinical outcomes were not different between the two groups at 3 years postoperatively.

**Conclusion:**

A higher preoperative ODI score may be a risk factor for postoperative ODI > 22 after surgery for LSS. It may also be associated with higher odds for improvements in the ODI score of > 12.8.

## Background

Surgery is not intended to be a first-line treatment for symptomatic lumbar spinal stenosis (LSS) because of the benign natural history of LSS [1, 2]. However, when conservative treatments are ineffective and pain and disability progressively worsen owing to LSS, surgical treatment such as decompression with or without fusion can be a reasonable treatment option. According to previous studies [2, 3], surgical outcomes of LSS tend to be favorable and the treatment effect is maintained for several years after surgery.

However, no consensus has been reached for defining favorable or successful surgical outcomes. Recently, the concept of minimal clinically important difference (MCID) has been used as a critical threshold to measure treatment effectiveness [4, 5]. The treatment effect of spinal surgery reaching the MCID may justify the decision for surgical treatment [4]. However, the MCID cannot reflect overall surgical outcomes in patients with LSS and only represents improvements in patient-reported outcomes (PROs) between the pre- and postoperative states. A recent study has reported that clinical improvement is not associated with patient satisfaction after surgery, while the postoperative Oswestry Disability Index (ODI) is associated with patient satisfaction with the surgical treatment [6].

A risk factor or predictor of surgical outcomes may be associated with the definitions of successful surgical outcomes. In general, two methods have been used to evaluate surgical outcomes: one is the absolute level achieved, for which PRO scores per se can be used postoperatively, and the other is the relative change after surgery, for which the MCID of the PRO scores can be used postoperatively. We hypothesized that risk factors for unfavorable surgical outcomes would be dependent on the definitions of unfavorable surgical outcomes. The aims of this study were: 1) to compare risk factors for each unfavorable surgical outcome according to the two different definitions of “unfavorable” surgical outcomes using either the MCID or PRO score method at 1 year postoperatively and 2) to compare patient clinical courses preoperatively to 3 years postoperatively between the favorable and unfavorable outcomes according to the two different definitions after surgery for LSS.

## Methods

### Study population

This study was within the framework of a longitudinal clinical study about surgical results for LSS. Our study was approved by our institutional review board (SNUBH-B-1802/448–112). Initially, 429 consecutive patients who underwent LSS from March 2012 to December 2015 were assessed for eligibility. Among these patients, those who fulfilled the following inclusion criteria were included: (1) age > 40 years, (2) a diagnosis of LSS and spine surgery for LSS scheduled at a tertiary institution, and (3) patients who had complete preoperative and follow-up data until at least 3 years postoperatively. In addition to a stenotic lesion of more than grade A on lumbar spine magnetic resonance imaging, the criterion for a diagnosis of LSS was the presence of one or more of the following symptoms, according to the grading scale developed by Schizas et al.: walking intolerance due to neurogenic claudication, pain/numbness/tingling sensation in the buttocks and lower extremities, and motor weakness, as well as bladder/bowel dysfunction [7, 8]. Grade A stenosis indicates that there is a clearly visible cerebrospinal fluid (CSF) inside the dural sac, but its distribution is inhomogeneous. Grade B stenosis indicates that the rootlets occupy the entire dural sac, but they can still be individualized. In grade C stenosis, no rootlets can be recognized and the dural sac demonstrates a homogeneous gray signal with no visible CSF signal. Grade D stenosis indicates that there is no posterior epidural fat or recognizable rootlets [8].

**Fig. 1:**
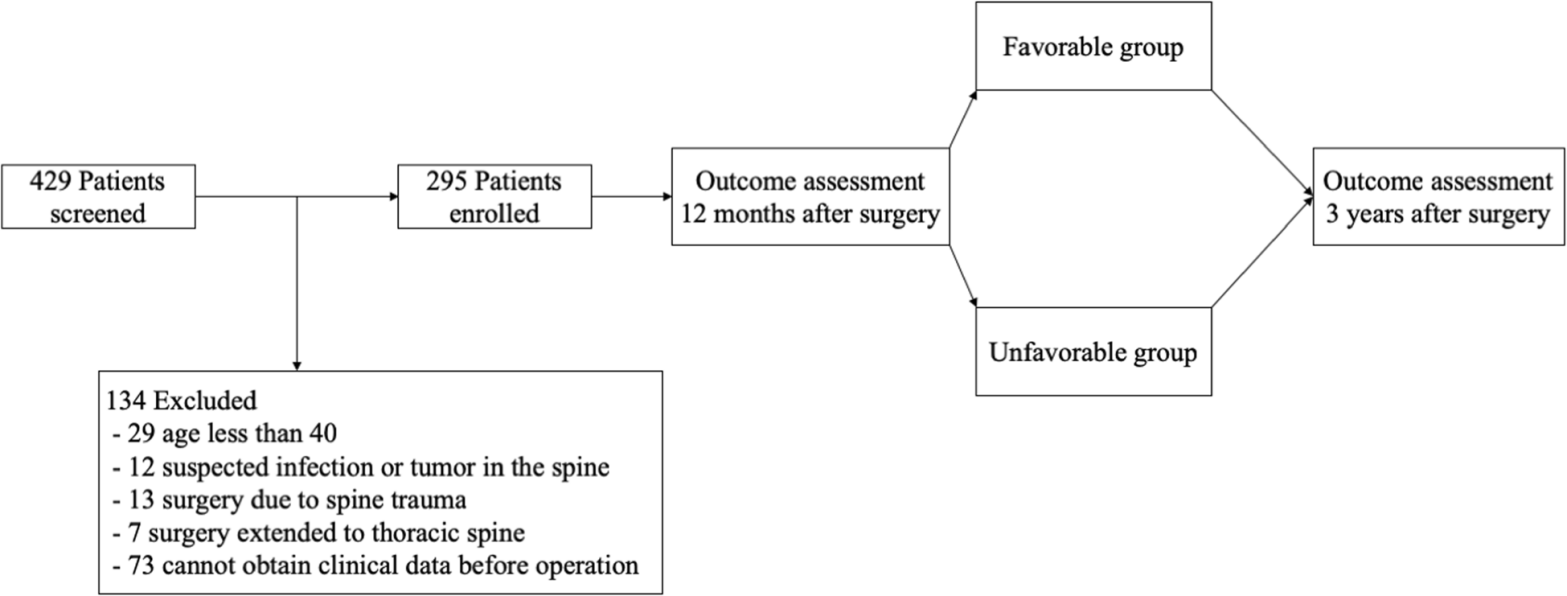
Flowchart of patient enrollment according to the PRO score and MCID methods. ODI, Oswestry Disability Index; PRO, patient-reported outcome; MCID, minimal clinically important difference

The exclusion criteria of this study were as follows: (1) surgery due to infectious, tumorous, or traumatic lesions; (2) surgery extended to the thoracic spine level; (3) unavailable or unclear preoperative clinical data; and (4) adult spinal deformity with coronal and/or sagittal imbalance. Of the 429 patients, 295 were enrolled in the study (Fig. 1).

### Study design

All prospectively collected data, as part of the routine care of the study patients, were de-identified and retrospectively reviewed. Baseline data, collected by a clinical research assistant otherwise blinded to the study, included sex, age, height, weight, body mass index (BMI), symptom duration, smoking history, educational attainment, medical comorbidity, level of stenosis, preoperative visual analog scale (VAS) scores for back and leg pain, and preoperative ODI score. The VAS scores for back and leg pain and ODI scores were reassessed at 1 and 3 years postoperatively by a clinical research assistant otherwise blinded to the study.

The included patients were divided into two groups, a favorable and unfavorable group, based on two different definitions for unfavorable outcomes at 1 year postoperatively. Preoperative risk factors of unfavorable outcomes were investigated based on the participant’s status according to each definition at 1 year postoperatively. After group allocation, surgical results in each group were reassessed at 3 years postoperatively.

### Definition of unfavorable outcomes after surgery

Surgical outcomes were evaluated by absolute outcomes and relative change achieved. For the former, the postoperative ODI score was used (the PRO score method), and for the latter, the MCID was used (the MCID method).

In the PRO score method, patients were stratified into the favorable (ODI ≤22) and unfavorable (ODI > 22) outcome groups at 1 year postoperatively, reflecting the level of well-being observed in healthy populations with no chronic lower back pain [9, 10]. In the MCID method, patients were stratified according to the MCID values for the ODI scores at 1 year postoperatively. A decrease of 12.8 points from the preoperative value was set as the cutoff for categorizing the “favorable” and “unfavorable” outcome groups [4].

The primary outcome was a comparison of risk factors for each unfavorable surgical outcome by two different definitions at 1 year postoperatively. The secondary outcome was a comparison of patients’ clinical courses preoperatively to 3 years after surgery between the favorable and unfavorable groups according to the two different definitions of surgical outcomes.

### Surgical procedures

One experienced orthopedic spine surgeon performed all surgical procedures. Decompressive surgery with or without fusion was performed for all patients. Simple decompression was the preferred method and was recommended for patients who did not have spondylolisthesis or had low-grade, static spondylolisthesis. The surgeon used a unilateral approach-bilateral decompression technique for all decompression cases. Additional fusion was performed if there was associated instability or a need for > 50% resection of the facet joints due to foraminal decompression, foraminal stenosis, degenerative spondylolisthesis grade > 2, scoliosis, or kyphosis. In the fusion portion of the surgery, the surgeon routinely performed a posterior interbody fusion technique (posterior or transforaminal lumbar interbody fusion) using the posterior approach.

### Variables

Baseline data, collected by a clinical research assistant otherwise blinded to the study, included sex, age, height, weight, BMI, symptom duration, smoking history, educational attainment, medical comorbidity, level of stenosis, preoperative pain intensity for back and leg pain, and preoperative ODI scores. BMI was measured after hospital admission for surgery, and the hospital’s standardized questionnaire was used for the assessment of any comorbidities. The VAS scores for back and leg pain and ODI scores were reassessed at 1 and 3 years postoperatively.

ODI (version 2.0; Mapi Research Trust, Lyon, France) is a self-administered questionnaire for measuring back-specific function. The questionnaire consists of 10 items, each with 6 response levels. Each item is scored from 0 to 5, and the total score (%) is converted to a scale of 0–100 [9]. Preoperative pain intensity was measured using a VAS score. The VAS for back and leg pain consists of a 10-cm line with “none” (0) at one end of the line and “disabling pain” (10) at the other end. Patients were asked to place a mark on the 10-cm line to represent their perceived level of back and leg pain. The measured distance (cm) from 0 to their mark was considered the VAS score.

### Statistical analysis

A descriptive analysis of each patient was performed, including preoperative ODI score, VAS scores for back and leg pain, and demographic data. To evaluate potential risk factors for unfavorable outcomes at 1 year postoperatively, various preoperative variables were initially evaluated in the univariate logistic regression analysis. Variables that were significantly associated with an unfavorable outcome at *P* <  0.20 in the univariate analysis were then evaluated in the multivariate logistic regression model, along with age and sex as significant risk factors for unfavorable outcomes. For the multivariate models, we anticipated a potential issue of collinearity between the variables and set an a priori rule to exclude variables with Pearson or phi correlation coefficients ≥0.30.

At both 1 and 3 years postoperatively, the ODI and VAS scores for back and leg pain were compared between the unfavorable and favorable groups according to the two different definitions using an independent *t*-test. All statistical analyses were performed using IBM SPSS Statistics software (version 20.0.0; IBM Corp., Armonk, NY USA). The level of significance was set at 0.05.

## Results

### Descriptive analysis of the study subjects

Between March 2012 and December 2015, 429 patients were assessed for their eligibility for inclusion in this study. Among these patients, 295 were enrolled in the study. At 1 year postoperatively, 147 and 103 patients were allocated to the unfavorable group according to the PRO score and MCID methods, respectively (Fig. 1). Table 1 presents baseline characteristics and preoperative clinical data of subjects in each group according to the PRO score and MCID methods. In the PRO score method, age and educational level were different between the favorable and unfavorable outcome groups, whereas other baseline demographic data were similar; the preoperative VAS score for back pain and ODI score were significantly lower in the favorable outcome group by the PRO score method. In the MCID method, all baseline demographic data of patients were similar between both groups; however, the preoperative VAS scores for back and leg pain and ODI score were significantly higher in the favorable outcome group by the MCID method. No significant differences in the type and level of surgery were found between the favorable and unfavorable groups by either criteria (Table 1).

**Table 1:** Univariate analysis for demographic and preoperative data between the favorable and unfavorable outcome groups in ***1-year postoperative*** according to the PRO score and MCID method

Characteristics	PRO score method (***patients***)			MCID method (***patients***)		
	Favorable Outcome (84)	Unfavorable Outcome (147)	*P* value	Favorable Outcome (128)	Unfavorable Outcome (103)	*P* value
**Age (years)**	65.2 ± 9.1	67.9 ± 9.0	0.026	66.63 ± 9.18	67.32 ± 9.11	0.566
**BMI (kg/m**^**2**^**)**	25.84 ± 4.16	25.36 ± 3.38	0.343	25.73 ± 4.03	25.30 ± 3.20	0.375
**Sex (Male**: **Female)**	38:46	33:114	< 0.001	38:90	33:70	0.404
**Type of surgery**						
Fusion	48	88	0.395	79	57	0.199
Decompression	36	59		49	46	
**Level of Surgery**						
1	72	121	0.216	107	86	0.470
2	12	23		20	15	
3	0	3		1	2	
**Educational level**						
Elementary school	9	45	0.005	27	27	0.190
Middle school	22	38		38	22	
High school	27	33		35	25	
University	25	29		28	26	
Unknown	1	2		0	3	
**Comorbidity**						
Hypertension	47	99	0.091	82	64	0.785
Diabetes	25	39	0.648	37	27	0.661
Ischemic heart disease	2	8	0.334	5	5	0.755
Kidney	1	6	0.427	4	3	1.000
Stroke	3	6	1.016	4	5	0.517
Liver disease	0	1	1.000	0	1	0.446
**Smoking history**						
Current smoker	7	9	0.322	9	7	0.885
Ex-smoker	14	16		16	14	
No smoking	63	119		102	80	
Unknown	0	3		1	2	
**Preoperative VAS for back pain**	5.48 ± 2.82	6.39 ± 2.49	**0.012**	6.52 ± 2.63	5.49 ± 2.57	**0.003**
**Preoperative VAS for leg pain**	6.79 ± 2.40	7.19 ± 2.27	0.196	7.34 ± 2.32	6.68 ± 2.29	**0.031**
**Preoperative ODI**	41.38 ± 15.44	48.28 ± 17.05	**0.002**	53.05 ± 16.47	36.74 ± 12.20	**< 0.001**

Mean ± standard deviation; ***BMI, body mass index; level of surgery, how many segments the surgery involved;*** PRO score method divided the patients into favorable and unfavorable outcome groups by ODI scores ≤22 and ODI scores > 22, respectively; MCID method divided the patients into more and less-improved outcome groups based on a decrease in ODI scores by greater than 12.8 or less; *VAS* Visual analog scale (***0–10***); *ODI* (***%***) Oswestry disability index (***0–100***)

### Risk factors for unfavorable outcomes by the PRO score and MCID methods

Each multivariate logistic regression model showed the odds ratio (OR) of risk factors for unfavorable outcome using the PRO score and MCID methods. In the PRO score method, female sex (vs. male sex; *P* = 0.011; OR: 2.340), elementary school attainment (vs. university attainment; *P* = 0.035; OR: 2.875), and higher preoperative ODI scores (*P* = 0.028; OR: 2.340) were associated with higher odds for an unfavorable surgical outcome (Table 2). In the MCID method, however, a preoperative higher ODI score was associated with higher odds (*P* < 0.001; OR: 0.920) for a favorable surgical outcome (Table 2).

**Table 2:** Multivariate logistic regression model of risk factors for unfavorable outcomes in the PRO score and the MCID method groups

Characteristics	PRO score method		MCID method	
	Odds Ratio(95% Confidence Interval)	P value	Odds Ratio(95% Confidence Interval)	*P* value
Age (years)	***1.032 (0.996–1.069)***	***0.080***	***1.026 (0.990–1.063)***	***0.166***
BMI (kg/m^2^)	***0.950 (0.875–1.030)***	***0.213***	***0.993 (0.908–1.085)***	***0.874***
***Sex (Female***)	***2.340 (1.211–4.520)***	***0.011***	***1.496 (0.708–3.162)***	***0.292***
Type of surgery (Decompression)			***1.161 (0.606–2.225)***	***0.652***
Educational level (vs. university)				
***Elementary school***	***2.875 (1.075–7.688)***	***0.035***	***1.299 (0.481–3.506)***	***0.606***
Middle school	***1.082 (0.456–2.566)***	***0.859***	***0.718 (0.283–1.819)***	***0.485***
High school	***0.859 (0.380–1.943)***	***0.715***	***0.892 (0.372–2.138)***	***0.798***
Hypertension	***1.387 (0.714–2.697)***	***0.334***		
**Preoperative ODI**	***2.340 (1.211–4.520)***	***0.028***	***0.920 (0.897–0.944)***	***< 0.001***

Mean ± standard deviation; PRO score method divided the patients into favorable and unfavorable outcome groups by ODI scores ≤22 and ODI scores > 22, respectively; MCID method divided the patients into more and less-improved outcome groups based on a decrease in ODI scores by greater than 12.8 or less; *VAS* Visual analog scale; *ODI* Oswestry disability index

### Changes of clinical outcomes in each group by the PRO score and MCID methods

**Fig. 2:**
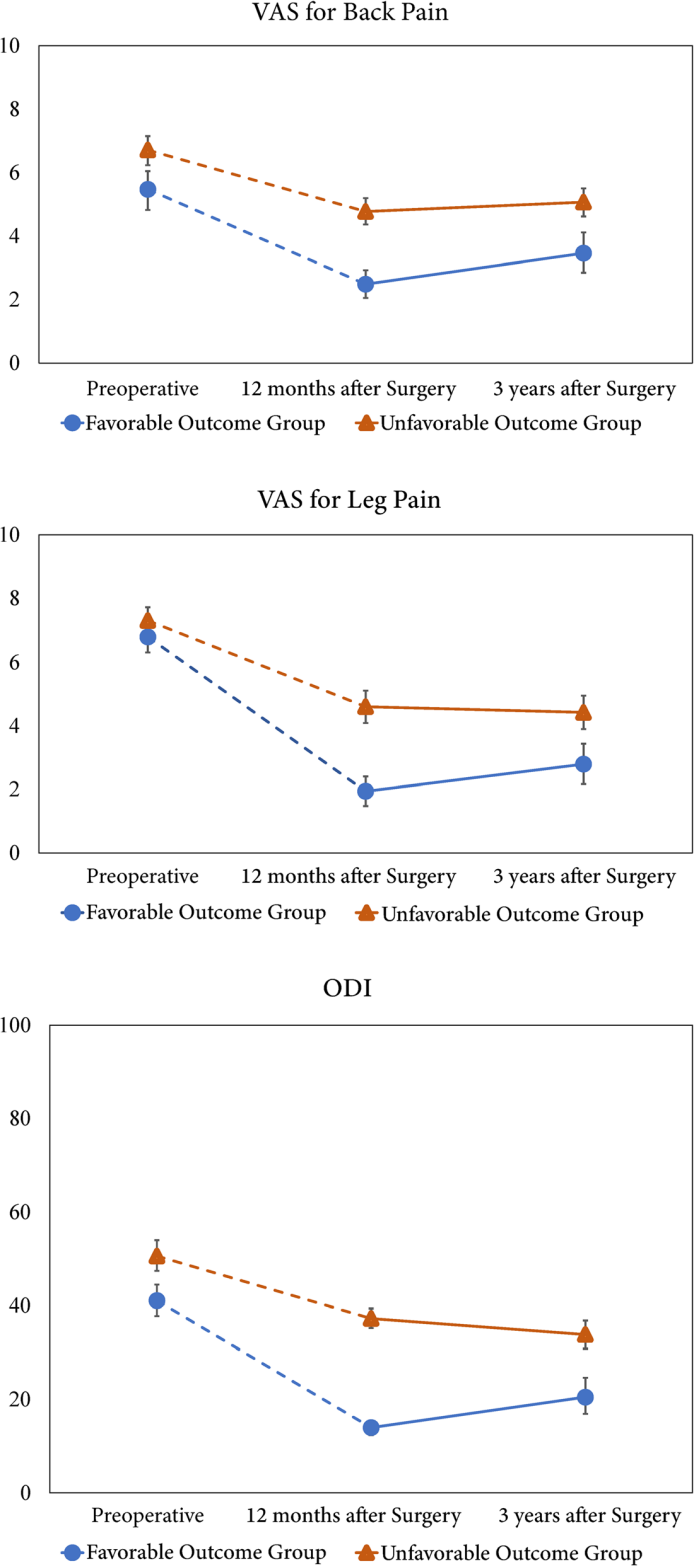
Treatment outcomes preoperatively and at 1 and 3 years postoperatively based on the PRO score method. Error bars indicate 95% confidence intervals. Top: VAS score for back pain; Middle: VAS score for leg pain; Bottom: ODI score. VAS, visual analog scale; ODI, Oswestry Disability Index; PRO, patient-reported outcome

In the PRO score method, from 1 to 3 years postoperatively, neither the favorable nor unfavorable outcome groups showed significant changes in the mean VAS scores for back and leg pain and ODI scores (Fig. 2). In addition, significant differences in the ODI and VAS scores for back and leg pain were observed at 1 and 3 years postoperatively between both groups (*P* < 0.001 for all variables; Fig. 2).

**Fig. 3:**
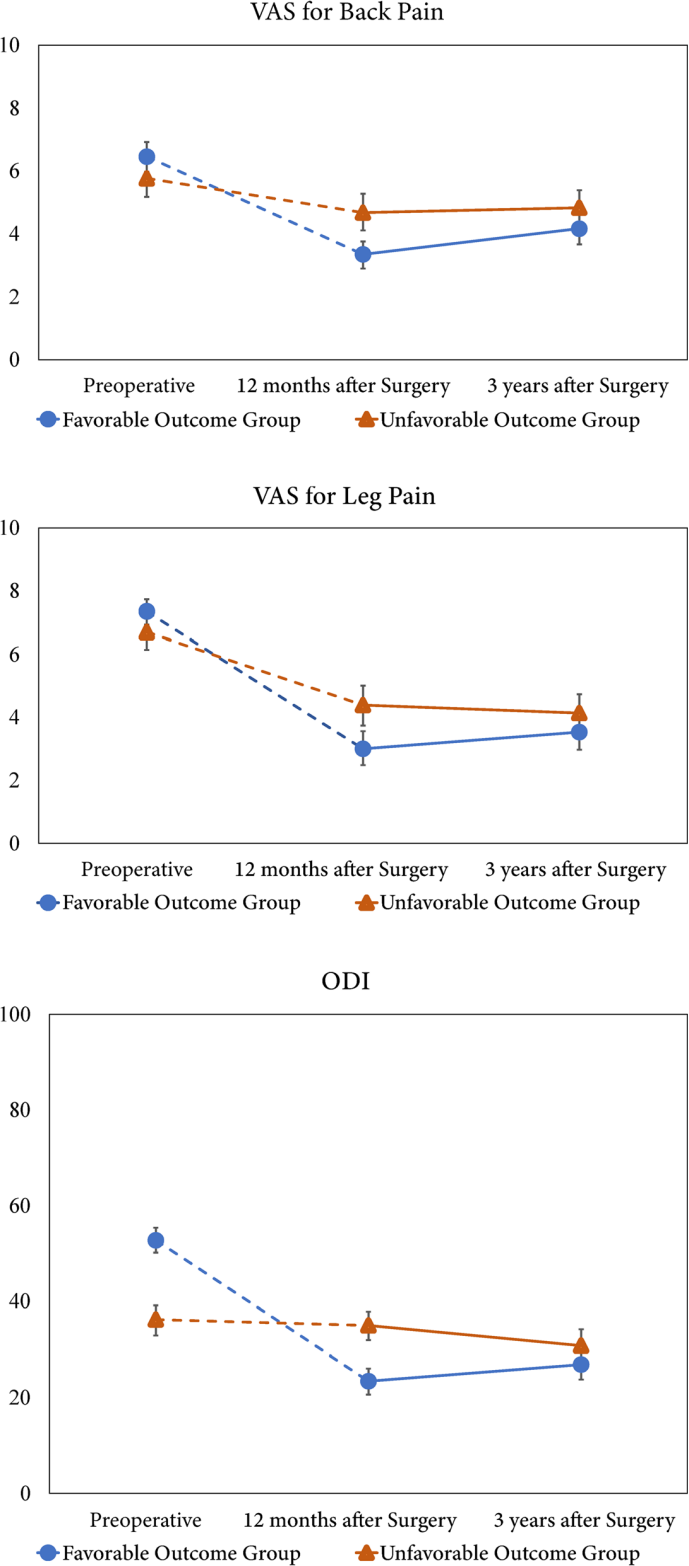
Treatment outcomes preoperatively and at 1 and 3 years postoperatively based on the MCID method. Error bars indicate 95% confidence intervals. Top: VAS score for back pain; Middle: VAS score for leg pain; Bottom: ODI score. VAS, visual analog scale; ODI, Oswestry Disability Index; MCID, minimal clinically important difference

In the MCID method, at 1 year postoperatively, the favorable outcome group demonstrated significantly lower VAS scores for back and leg pain and ODI scores than the unfavorable outcome group (*P* < 0.001, *P* = 0.002, and *P* < 0.001, respectively). However, at 3 years postoperatively, no significant differences in the VAS scores for back and leg pain and ODI scores were found between the favorable and unfavorable groups (*P* = 0.122, 0.170, and 0.152 for the VAS score for back pain, VAS score for leg pain, and ODI score, respectively; Fig. 3).

## Discussion

The present study demonstrated that risk factors for unfavorable surgical outcomes in patients with LSS were dependent on the definitions of unfavorable surgical outcomes. When the surgical outcome was determined on the absolute score of the postoperative ODI, a preoperative high ODI score was associated with higher odds for unfavorable surgical outcomes (with a still-higher postoperative ODI score > 22), whereas when the surgical outcome was judged on the basis of relative changes in the ODI score between the pre- and postoperative states, a higher preoperative ODI score was associated with higher odds for a favorable surgical outcome (improvement of the ODI score > 12.8).

These results are in line with those of previous studies regarding surgical outcomes for LSS, which reported that severe preoperative symptoms and increased ODI scores were associated with less favorable postoperative outcomes [11–14]. In addition, the present study demonstrated that an increased preoperative ODI score was associated with higher odds for improvement of the ODI score measured using the MCID method. A previous study by Soriano et al. supports this finding, in which higher preoperative ODI scores were associated with greater ODI improvements between the preoperative and 1-year postoperative states [12]. Therefore, the present results suggest that preoperative disability would serve as a predictor or risk factor for surgical outcomes, depending on the definition of favorable surgical outcomes. A previous study agreed with these findings, in which even though women had more-severe preoperative symptoms and disabilities than men, they had higher odds for improvement than men [15]. In this study, likewise, female sex was not associated with unfavorable outcomes on the basis of the MCID method but was associated with unfavorable outcomes on the basis of the PRO method.

The multivariate logistic model showed that female sex, low educational level (graduation from elementary school), and an increased preoperative ODI score were associated with higher odds for an unfavorable outcome in terms of the PRO score method. Many previous studies advocated that low educational attainment was related to negative perceptions about preoperative disability [16–18]. Therefore, the first plausible explanation may be that a low educational level is associated with poor psychological coping with surgery and negative interpretations of postoperative symptoms [12]. A previous study also demonstrated that patients with higher educational levels and optimistic preoperative expectations had more favorable postoperative outcomes [12]. From the perspective of MCID, however, a low education level was not associated with higher odds for unfavorable outcomes. This may be explained by the fact that a low education level is related to high preoperative levels of pain and disability, which may result in greater ODI improvement between the preoperative and 1-year postoperative states [12].

In the PRO score method, from 1 year until at least 3 years postoperatively, neither the favorable or unfavorable outcome groups showed significant changes in the mean VAS scores for back or leg pain or ODI scores. Therefore, the unfavorable outcome group, which demonstrated inferior surgical outcomes at 1 year postoperatively, still showed higher ODI and VAS scores for back and leg pain at 3 years postoperatively than the favorable outcome group. A previous study reported similar findings to the present results [19], in which the PRO scores for functional disability and pain severity at 12 months accurately reflected those at 24 months after LSS, irrespective of the surgical procedure.

The present study has limitations that should be acknowledged. First, a single surgeon performed the surgery and collected clinical data of all patients enrolled; therefore, generalization of clinical findings may not be appropriate. However, we chose the commonly used surgical indications for LSS, and standardized surgical methods were used for both the decompression and instrumented fusions. Second, this observational study was not conducted in a prospective manner; prospective postoperative observation would have limited the potential selection bias by retrospective analysis in the present results. Third, the ODI score was used to define favorable surgical outcomes as only one construct. In fact, there are other factors that could affect the reporting of favorable surgical outcomes from the perspective of patients, including emotional aspects, functional recovery such as walking and standing ability, and symptom recovery such as improvement of weakness and/or pain-discomfort [20]. Related to this limitation, the ODI score was used for both the definition and predictive factor of surgical outcomes. This is a methodological limitation of this study.

## Conclusion

In conclusion, this study identified that risk factors for unfavorable postoperative outcomes depend on the definitions of unfavorable outcomes. Increased preoperative ODI scores are a risk factor for a postoperative ODI > 22 after surgery for LSS, whereas it is associated with higher odds for improvements in the ODI score of > 12.8.

## Data Availability

The datasets generated and/or analyzed during the current study are available from the corresponding author on reasonable request.

## Abbreviations

LSS: Lumbar spinal stenosis
MCID: Minimal clinically important difference
PROs: Patient-reported outcomes
ODI: Oswestry disability index
BMI: Body mass index
VAS: Visual analog scale
OR: Odds ratio
